# 4D pathology: translating dynamic epithelial tubulogenesis to prostate cancer pathology

**DOI:** 10.1111/his.15354

**Published:** 2024-10-20

**Authors:** Hridya Harikumar, Martin E van Royen, Geert JLH van Leenders

**Affiliations:** ^1^ Department of Pathology, Erasmus MC Cancer Institute University Medical Centre Rotterdam the Netherlands

**Keywords:** cell polarity, cribriform, growth pattern, lumen formation, prostate cancer, tubulogenesis

## Abstract

The Gleason score is the gold standard for grading of prostate cancer (PCa) and is assessed by assigning specific grades to different microscopical growth patterns. Aside from the Gleason grades, individual growth patterns such as cribriform architecture were recently shown to have independent prognostic value for disease outcome. PCa grading is performed on static tissue samples collected at one point in time, whereas *in vivo* epithelial tumour structures are dynamically invading, branching and expanding into the surrounding stroma. Due to the lack of models that are able to track human PCa microscopical developments over time, our understanding of underlying tissue dynamics is sparse. We postulate that human PCa expansion utilizes embryonic and developmental tubulogenetic pathways. The aim of this study is to provide a comprehensive overview of developmental pathways of normal epithelial tubule formation, elongation, and branching, and relate those to the static microscopical PCa growth patterns observed in daily clinical practise. This study could provide a rationale for the discerned pathological interobserver variability and the clinical outcome differences between PCa growth patterns.

## Graphical abstract

Rethinking prostate cancer growth patterns in the context of epithelial tubule formation, elongation, and branching.

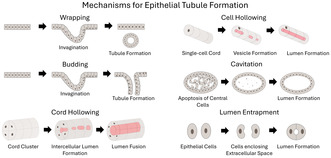

**Abbreviations**
ABP: apical‐basal polarity
AJ: adherens junction
BARW: branching and annihilating random walks
BMP: bone morphogenetic protein
CCM: collective cell migration
CE: convergent extension
CRB: crumbs
EMT: epithelial‐mesenchymal transition
GP: Gleason patterns
H&E: haematoxylin and eosin
MDCK: Madin‐Darby Canine Kidney
MET: mesenchymal‐epithelial transition
PAR: partition defective
PCa: prostate cancer
PCP: planar

## Introduction

The Gleason score is the globally used grading system for prostate cancer (PCa). It plays an essential role in estimating patients' prognosis and therapeutic decision‐making. The Gleason score is assessed by adding the two most common Gleason patterns (GP) on radical prostatectomy specimens or the most common and highest patterns on biopsies. Men with a biopsy Gleason score of 6 (3 + 3) are candidates for surveillance, while those with higher scores are generally offered active treatment.

PCa grading is performed on haematoxylin and eosin (H&E)‐stained tissue sections. GP1, 2, and 3 consist of malignant, well‐delineated tubules with well‐formed round to oval lumens.^1^ GP4 encompasses poorly‐formed, fused, cribriform, and glomeruloid structures, while GP5 is characterized by cords, single cells, solid fields, or the presence of comedonecrosis. A major shortcoming of the Gleason grading system is its substantial interobserver variability. For example, a study conducted among 337 European pathologists showed only 56% agreement on the Gleason score.^2^ Recently, individual growth patterns, in particular a cribriform pattern, were recognized to have independent prognostic value and are now included as eligibility criteria for active surveillance in Gleason score 7 (3 + 4) patients.^3^ An overview of PCa growth patterns is provided in Table 1.

**Table 1:** Overview of established prostate cancer growth patterns

Growth pattern	Morphology	H&E example
GP3	2D well‐delineated round to ellipsoid glands 3D tubules with sporadic branching and interconnection	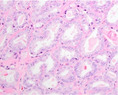
GP4 Poorly‐formed	2D small glands with irregular outline 3D small tubules with frequent branching, interconnections and blind ends	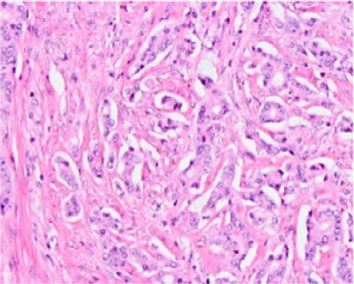
Fused	2D closely packed glands with interconnections and subtle interglandular fibrovascular stromal cores 3D tubules with frequent interconnections at close distance	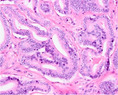
Glomeruloid	2D dilated glands with glomerulus‐like epithelial protrusions 3D dilated tubules with intraluminal epithelial protrusions either with or without stroma core	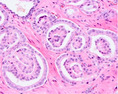
Cribriform	2D contiguous epithelial sheet with intercellular lumens and most epithelial cells not contacting adjacent stroma 3D large serpentine contiguous epithelial proliferation with intercellular lumens and most cells not contacting stroma	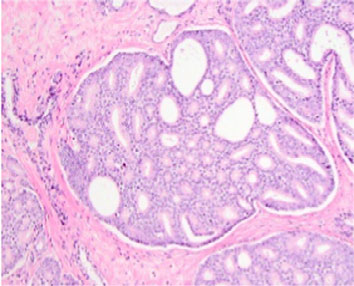
GP5 Cords	2D few‐cells‐thick epithelial cords without intercellular lumens 3D one to three cells' thick meshwork of epithelial cells without intercellular lumens	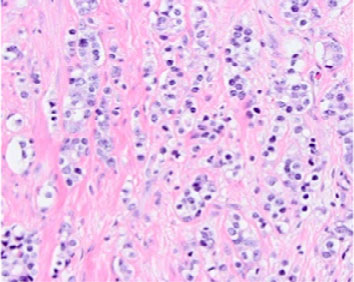
Single cells	2D single epithelial cells with or without intracytoplasmic vacuole 3D not determined	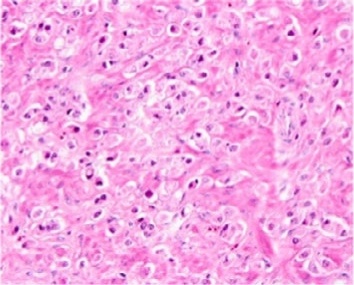
Solid	2D contiguous epithelial sheet without intercellular lumens and most epithelial cells not contacting adjacent stroma 3D large serpentine contiguous epithelial proliferation without lumens and most cells not contacting stroma	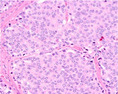

2D, two‐dimensional; 3D, three‐dimensional; GP, growth pattern.

Gleason grading is entirely based on the assessment of 4–5 μm cross‐sections of tissue samples, resulting in a two‐dimensional representation of the disease. Recently, upon investigating the actual underlying 3D architecture of separate growth patterns, we recognized two PCa growth pattern families: (i) those in which all tumour cells contact surrounding stroma including GP3, poorly‐formed and fused GP4, single cells and cords GP5, and (ii) those in which most tumour cells do not contact adjacent stroma including cribriform GP4 and solid GP5 either with or without comedonecrosis.^4^ The growth patterns within each family demonstrate gradual morphological and spatial continuity. For instance, 3D GP5 cords frequently form intercellular lumens compatible with poorly‐ formed GP4, suggesting a morphogenetic continuity between these patterns. Clinically, the natural existence of gradual morphological transitions between patterns would provide one explanation for the substantial interobserver variability in PCa grading.

Pathological assessment is typically done on static tissue structures fixed at one point in time, whereas growth patterns, in reality, are dynamic structures. However, getting insight into PCa structural dynamics is hampered by the lack of representative models that can track epithelial tubule development in time. Basic knowledge of embryonic and developmental mechanisms involved in normal tubule formation, elongation, and branching may shed light on pathological tumour morphogenetic processes and facilitate the comprehensive integration of PCa histopathology, molecular phenotype, and clinical behaviour. The aim of this review is (i) to provide an overview of basic developmental mechanisms involved in the formation, elongation, and branching of normal tubules, and (ii) to interpret static pathological PCa growth patterns in the light of being part of dynamically evolving, four‐dimensional, epithelial structures.

## Key molecular pathways and structural elements of normal tubular epithelial maintenance

The following section briefly discusses the key structural elements and molecular pathways involved in normal cell connection and polarity, which are prerequisites for epithelial tubule maintenance and dynamics.

### Cell junctions

**Figure 1:**
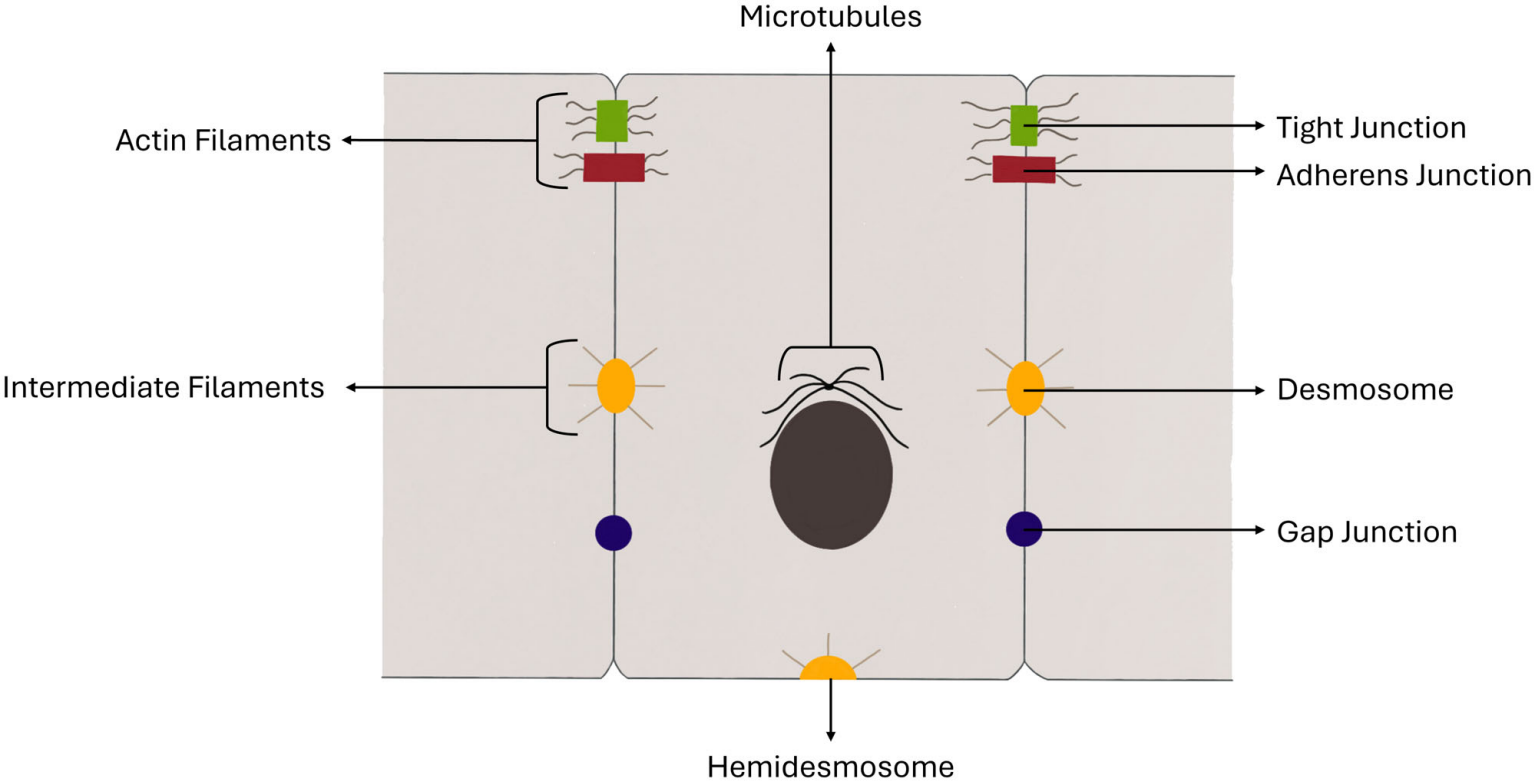
Schematic overview of cell junctions in epithelial cells. The five junctions—tight junctions, adherens junctions, desmosomes, hemidesmosomes, and gap junctions—are indicated. Actin filaments are tethered to tight and adherens junctions, while intermediate filaments are attached to the desmosomes and hemidesmosomes. The microtubules arrange themselves around the nucleus and are involved in maintaining cell shape and motility. [Color figure can be viewed at wileyonlinelibrary.com]

Epithelial cells form the functional unit of a tubule and can be classified according to their number of layers (simple, stratified, or pseudo‐stratified) and their shape (cuboidal, columnar, or squamous).^5^ Three cell membrane domains can be recognized in mature tubular epithelium: the apical side facing the lumen, the lateral sides in close contact with neighbouring cells, and the basal side anchoring the cell to the extracellular matrix. Mammalian epithelia are connected to neighbouring cells at the lateral sides through tight‐, adherens‐ and gap‐junctions, as well as desmosomes (Figure 1).^6^, ^7^ Hemi‐desmosomes connect epithelial cells to the adjacent extracellular matrix primarily via integrins.

Tight junctions (TJ) are the cell–cell contacts located most apically near the tubular lumen and have both a “fence” and “gate” function. TJ prevent the mixing of apical and lateral membranous lipids and other molecules, and form a barrier between luminal and intercellular compartments. TJ are molecular complexes that are mainly composed of claudins, occludins, and zona occludens (ZO) proteins. Three‐dimensionally, mature prostate luminal epithelial cells have a hexagonal or, less frequently, a pentagonal shape.^8^ Tricellular TJ containing the protein tricellulin secure the tightening of their neighbouring cells at their central contacting point.^9^

Adherens junctions (AJ), situated just below the TJ, are responsible for strong intercellular adhesion and are composed of nectins, cadherins, and catenins.^6^, ^10^ TJ and AJ connect to the intracellular flexible actin skeleton, form band‐like structures between epithelial tubular cells, and establish cell polarity by delineating the apical and lateral membranes. Beneath AJ, desmosomes form button‐like intercellular connections anchored to the stiff intracytoplasmic intermediate filament cell skeleton, that is, cytokeratins in tubules, which supports the 3D epithelial cell architecture. Additionally, the microtubules, which are anchored at centrosomes near the nucleus, contribute to cell polarization, migration, and intracellular trafficking.^11^ Finally, button‐like gap junctions allow for intercellular transfer of molecules, are composed of connexins, and do not connect to the intracytoplasmic cell skeleton.^6^, ^10^

### Apical‐basal polarity

**Figure 2:**
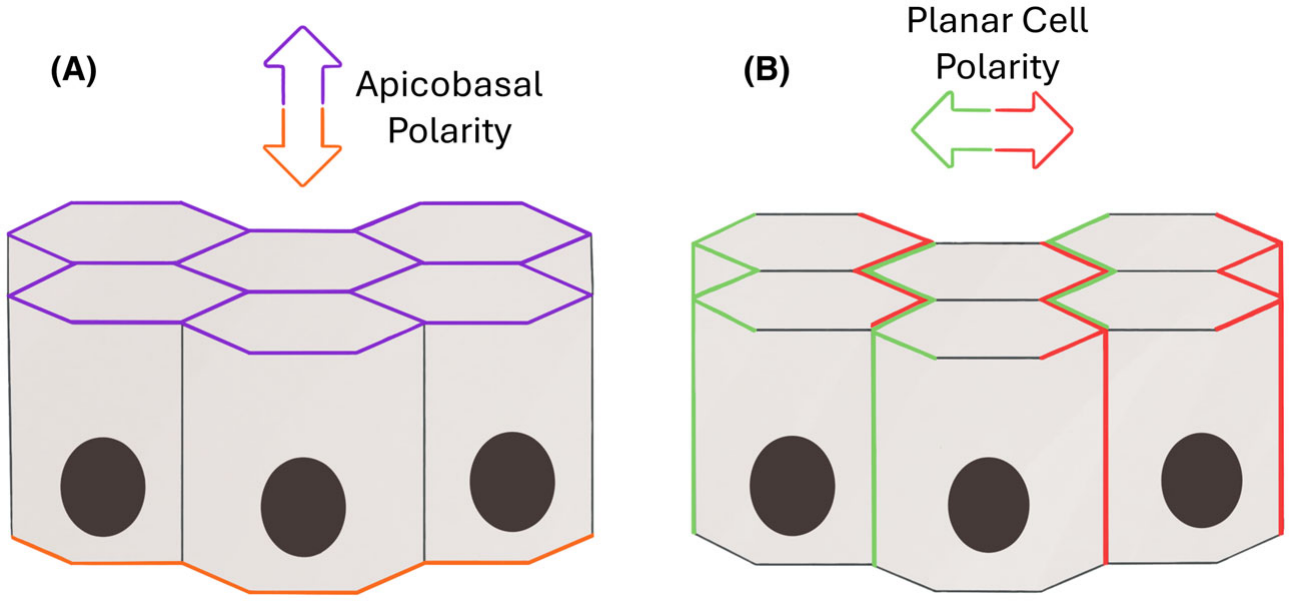
Apicobasal (**A**) and planar (**B**) cell polarity. (**A**) The apical surface is marked purple and the basal side brown. (**B**) Planar cell polarity is perpendicular to the apicobasal axis; the cellular front is labelled green and the rear red. [Color figure can be viewed at wileyonlinelibrary.com]

Apical‐basal polarity (ABP) is crucial to the morphology and function of epithelial cells lining tubules (Figure 2A). The spatial location and function of intercellular and cell‐matrix junctions are indispensable for establishing ABP. Cell polarization is initiated and regulated through several mechanisms. The basal membrane forms an initial cue to initiate polarization via β1‐integrin binding and signalling. The interaction of β1‐integrins with Rho‐GTPase Rac1 allows for basal lamina assembly, which acts as a reference for the apical‐basal orientation of cells.^12^ Polarization can also be initiated through interactions with neighbouring cells via AJ.^13^ AJ act as reference points for the asymmetric distribution of polarity complexes on the apical or basal side of epithelial cells.

Internal cell polarity is initiated and maintained by the interplay of three conserved polarity complexes, that is, the apical Partition defective (PAR) and Crumbs (CRB) complexes, and the basolateral Scribble (SCRIB) complex.^14^ These polarity complexes are crucial for the formation and maintenance of TJ and AJ. CRB3 also plays a major role in the formation of the apical membrane, substantiated by a considerable number of epithelial tubulogenetic defects leading to death in CRB3 knockout mice.^15^ Knockout of SCRIB in the prostate epithelium in mice led to prostate hyperplasia and prostate intraepithelial neoplasia (PIN). In contrast, SCRIB downregulation has been associated with adverse disease outcomes in clinical PCa.^16^

### Planar cell polarity

While ABP defines the basal and apical side of a tubular cell, planar cell polarity (PCP) determines the organization of cells along the plane of tissues (Figure 2B).^17^ PCP is oriented perpendicular to the apicobasal axis and distinguishes the front, side, and back of tubular epithelial cells.^18^ By opposing the localization of a set of transmembrane PCP receptors, such as Frizzled, at the front and back, epithelial cells provide information on their orientation to neighbouring cells. Upon stimulation of the Frizzled receptors by Wnt‐ligands, downstream effectors such as RhoA GTPase and cdc42 induce cytoskeletal rearrangements and organization.^19^, ^20^, ^21^ These small GTPases can facilitate cell migration by controlling the polymerization of actin at the anterior side and the contraction of actomyosin filaments at the posterior side of the cell.^22^ There are several interactions between ABP and PCP proteins, which implies these pathways are not mutually exclusive and may be required for each other's establishment.^17^ Downstream PCP signalling and SCRIB interaction are, for instance, important for mitotic spindle cell orientation parallel to the plane of the epithelium. Although investigations of ABP and PCP complexes have mainly been performed in cell lines and embryogenic *Drosophila* models,^23^ disruption of E‐cadherin and SCRIB in a mouse model led to the randomization of mitotic spindle orientation, epithelial hyperplasia, and invasive adenocarcinoma.^16^, ^23^ Little is yet known about the function of polarity complexes in human prostate homeostasis and carcinogenesis.

## Epithelial tubule dynamics in normal organogenesis

From a developmental point of view, epithelial tubule formation consists of four phases: (i) primitive tubule formation, (ii) tubule elongation, (iii) lumen expansion, and (iv) tubular branching.^24^ In the following, we will describe these stages and link normal developmental tubule dynamics to histopathological PCa growth patterns. Although some morphogenetic events have primarily been described in nonepithelial tissues, we include them for completeness.

## Primitive tubule formation

Primitive tubule formation mechanisms can be classified based on their cell polarity or the origin of their lumen. At least five mechanisms for normal primitive tubule formation can be recognized, that is, wrapping and budding, cord hollowing, cell hollowing, cavitation, and lumen entrapment, which are described in detail below.

### Wrapping and budding

**Figure 3:**
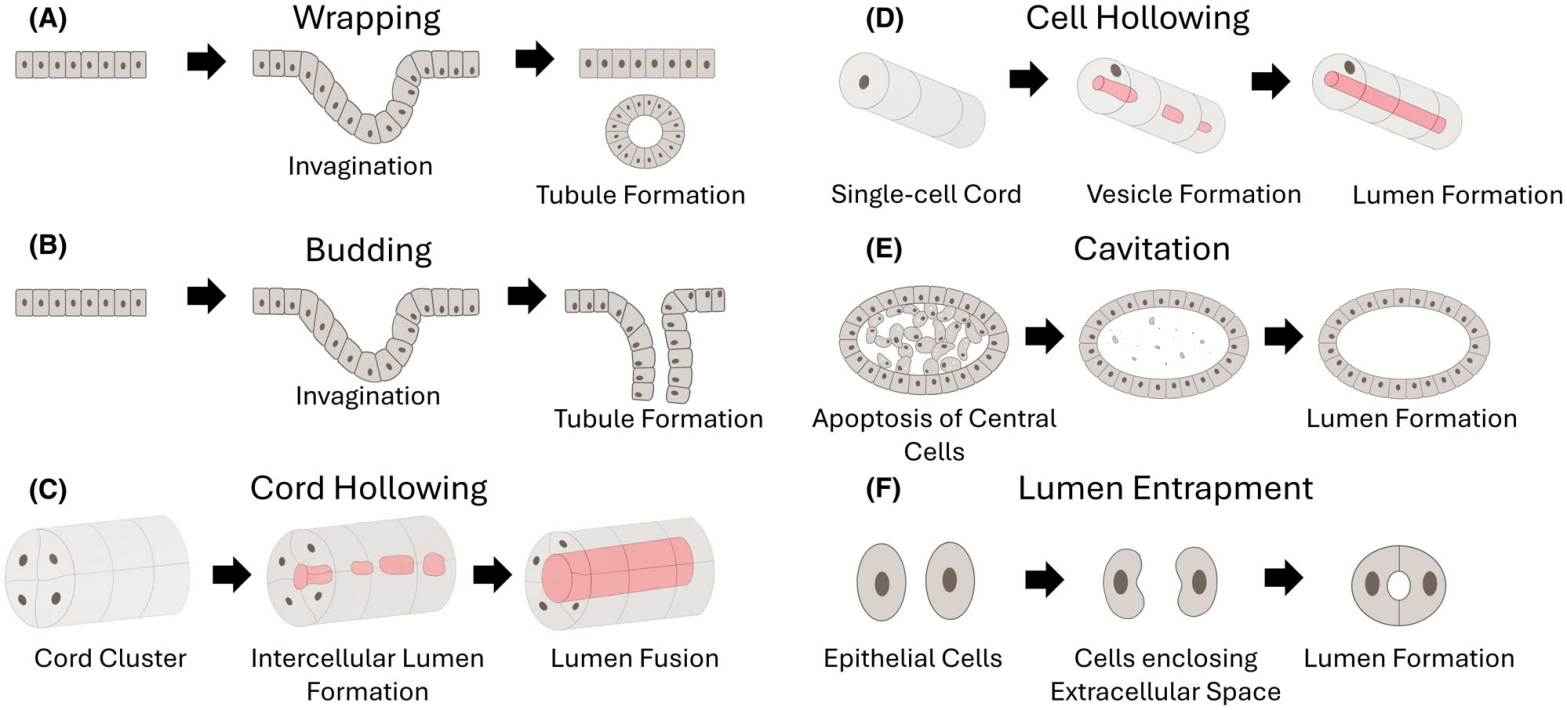
Mechanisms for primitive tubule formation. (**A**) Wrapping involves the invagination of an epithelial placode into stroma with eventual fusion of the lateral cells to form a lumen. (**B**) Budding is similar to wrapping, where an invagination occurs from an epithelial placode but the lateral cells remain in contact with the surface epithelium. (**C**) Cord hollowing is established by epithelial cell polarization, formation of intercellular lumens, and luminal coalescence. (**D**) In cell hollowing, intracytoplasmic lumens are formed and might finally fuse with cell membranes. (**E**) Cavitation involves apoptosis of central cells in a solid cell cluster. (**F**) In lumen entrapment two cells enclose extracellular space to form a lumen. [Color figure can be viewed at wileyonlinelibrary.com]

During wrapping and budding, tubule formation originates from a preexistent columnar epithelial placode.^24^, ^25^ Due to the apical constriction of the actomyosin skeleton under the influence of Rho‐associated kinases (ROCKs), the shape of epithelial cells changes from cylindrical to pyramidal, which is the first step towards invagination into the underlying stroma. In wrapping, the polarized lateral cells of the invagination fuse at the top just beneath the originating placode and so fully enclose a secondary lumen (Figure 3A).^24^, ^25^, ^26^ In budding, the lateral cells remain contiguous with the original epithelial placode and the lumen can be further extended via cell migration or cell proliferation (Figure 3B).^24^, ^25^ As a result, wrapping results in a tubule parallel to the primary epithelial placode and budding in a tubule perpendicular to the original placode. Wrapping can be observed during embryonic neural tube formation in mammals, while budding is involved in branching morphogenesis, such as in lung development.^25^ Budding is also an essential mechanism in normal prostate branching organogenesis and is, among others, regulated by Wnt‐ and bone morphogenetic protein (BMP) pathways.^27^, ^28^ Reactivation of budding morphogenesis has been linked to both benign prostate hyperplasia and PCa.^29^, ^30^ Using detailed immunohistochemical analysis, Nagle *et al*. hypothesized that PCa invasion is initiated by luminal cell budding out of PIN at locations where circumventing basal cells are absent.^31^ In line with this hypothesis, branching in GP3 PCa could also result from epithelial budding.^4^

### Cord hollowing

During embryogenesis, tubule formation can also originate from mesenchymal cells after undergoing mesenchymal‐epithelial transition (MET).^10^ MET generally involves the upregulation of E‐cadherin and the formation of AJ. During embryonic cord hollowing, groups of mesenchymal cells arranged in cord clusters are first formed (Figure 3C).^24^, ^25^, ^32^ These clustered cells begin to form primitive cell–cell junctions, which initiate the epithelial polarization process and formation of small intercellular apical lumina. The so‐called midbody model can explain the formation of intercellular apical lumens in previously unpolarised cells. The mitotic midbody serves as a polarity cue during cell division, forming the landmark for endosomal apical membrane fusion. Subsequently, the intercellular space is demarcated by the formation of TJ and the recruitment of apical proteins such as CRB3.^33^ Unlike wrapping and budding, in cord hollowing the cells undergo polarization due to external cues, and there is *de novo* lumen formation.^25^, ^26^, ^34^ This mechanism can be observed during mammalian dorsal aorta formation and in MDCK cells *in vitro*.^10^, ^25^ Cord hollowing is also involved in the formation of comma‐ and S‐shaped bodies of the human proximal nephron from caudal mesonephros, and these structures eventually fuse with the Wolffian ducts during embryogenesis.^35^, ^36^, ^37^

While cord hollowing has mostly been studied during normal embryogenesis, histopathological GP5 cords show a remarkable resemblance to this developmental process. In a study investigating 3D architecture, we found that some GP5 cords have minute intercellular lumina, which gradually transition to cell structures with clearly visible intercellular lumina being classified as poorly‐formed GP4.^4^ Based on this 3D observation and the established developmental sequence of events, we speculate that GP5 cords might be a precursor of some poorly‐formed GP4 tubules. Clinicopathologically, this could explain the substantial interobserver variability of distinguishing both patterns and their comparable low risk of metastatic progression in the absence of invasive cribriform and intraductal carcinoma.^2^, ^38^

### Cell hollowing

Similar to cord hollowing, during normal embryogenic cell hollowing, a cord of single mesenchymal cells is first formed (Figure 3D).^24^ After epithelisation, the cells then form intracellular vesicular structures called “vacuolar apical compartments” (VACs), which, upon exocytosis, can give rise to intercellular lumen formation.^10^, ^24^ Similar to cord hollowing, cell hollowing results in *de novo* lumen formation in normal cells.^26^ In clinical PCa, intracytoplasmic vacuolisation can sometimes be observed in GP5 single cells and is associated with loss of E‐cadherin.^39^ Genomic *CDH1* alterations with loss of E‐cadherin expression are observed in several tumour types such as lobular mammary, plasmacytoid urothelial, and diffuse gastric carcinoma, and result in pathological morphologies similar to single‐cell GP5 PCa. Here, it is important to stress the distinction between (i) physiological and reversible E‐cadherin downregulation as observed in poorly‐formed GP4, and (ii) irreversible E‐cadherin loss due to genomic alterations as seen in single‐cell GP5.

### Cavitation

During normal embryogenic cavitation, a solid cylindrical mass of mesenchymal cells is first formed^24^, ^25^ (Figure 3E). Then the central cells of the mass undergo apoptosis to form a lumen while the outer cells epithelialise.^24^ Similar to the other hollowing mechanisms, cavitation involves the polarization of cells due to external cues and *de novo* formation of a lumen.^25^, ^26^, ^34^ This process is known to be utilized in the formation of mammary glands and mammalian salivary glands.^10^, ^25^ As far as we know, cavitation has not been observed in the context of prostate tubulogenesis.

### Lumen entrapment

For completeness, this lumen formation process happens when two migrating cells entrap extracellular space (Figure 3F). The opposing edges of the two cells are adjoined, while their opposing centres are repelled. Similar to wrapping and budding, lumen entrapment involves polarized cells that encircle a preexistent luminal extracellular space.^25^, ^26^ Lumen entrapment is observed during embryonic heart formation in *Drosophila*.^25^, ^40^

## Tubule elongation in normal organogenesis

Tubule elongation occurs through changes in cell shape and arrangement using cell proliferation and recruitment.^5^ Several mechanisms are involved in the expansion of tubules, including epithelial‐mesenchymal transition (EMT), collective cell migration, and cell intercalation. These processes have frequently been investigated as separate mechanisms of epithelial invasion, but putatively form the extremes of a migratory morphological continuum.^41^

### Epithelial‐mesenchymal transition

**Figure 4:**
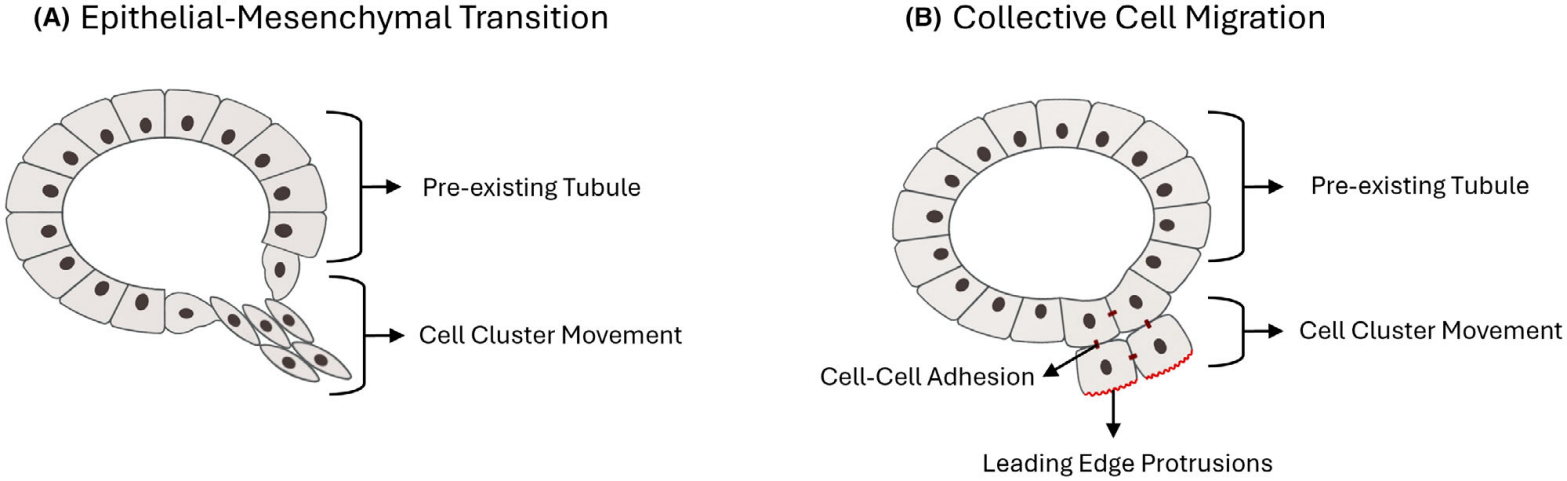
Cell migration mechanisms. (**A**) In epithelial‐mesenchymal transition (EMT) cell clusters migrate from a preexisting tubule by loosening intercellular contacts. (**B**) In collective cell migration, cell clusters move while leaving cell–cell adhesions intact. [Color figure can be viewed at wileyonlinelibrary.com]

EMT is one of the best‐studied and well‐known mechanisms for tubule elongation and cell migration in normal development and pathological processes (Figure 4A). Herein, epithelial cells lose or downregulate epithelial markers such as E‐cadherin while they acquire or upregulate mesenchymal markers such as N‐cadherin, OB‐cadherin, and vimentin. In general, EMT due to downregulation of E‐cadherin is considered a reversible process, which can be followed by MET, reestablishing epithelial maturation and polarization.

Complete EMT, with the formation of spindle cells and complete loss of epithelial cell markers, is not observed in clinical PCa except for its rare sarcomatoid subtype. More frequently, epithelial cells undergo partial EMT, combining features of both epithelial and mesenchymal cells; for example, by coexpression of cytokeratins and N‐cadherin. Kolijn *et al*. found that poorly‐formed GP4, which is characterized by its irregular and jagged morphology, is enriched for tumour cells with a partial EMT phenotype, expressing N‐cadherin with concomitant downregulation of E‐cadherin.^42^ Since EMT is mostly considered a reversible process, it is tempting to speculate that some poorly‐formed GP4 structures could mature towards well‐defined GP3 tubules.

#### Collective cell migration

While EMT mainly refers to a dichotomous state of single cells, collective cell migration (CCM) refers to the movement of a group of cells that remain stably connected via cell adhesion.^43^ Generally, the cells that partake in collective cell migration are of two types: leader and follower cells. The cells at the leading edge can respond to environmental cues such as growth factors and chemokines, and become appropriately polarized along the direction of migration. Lamellipodia and filopodia extend from these cells, which help in directional migration. The follower cells are dragged along due to their cell–cell adhesion connections to the leader cells (Figure 4B).

Based on various studies involving MDCK cells cultured in a 3D matrix, N‐cadherin has been established to initiate CCM through both cell–cell adhesion and direct interaction with cell migration pathways.^44^ Knockdown of N‐cadherin in these cells prevented them from forming clusters and migrating.^45^ Besides cell–cell adhesion, the establishment of front‐rear polarity is essential for CCM and is regulated by Rho GTPases.^46^ The asymmetric localisation of Rac1 at the leading edge and RhoA at the trailing edge primarily maintain this front‐rear polarity.

### Cell intercalation

**Figure 5:**
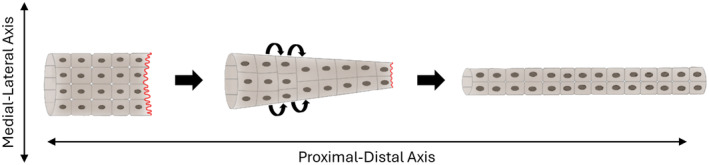
Convergent extension (CE) in tubules. Collective cell migration (CCM) occurs along the proximal‐distal axis where the leading cells are indicated with red cellular extensions. Cell intercalation occurs along the medial‐lateral axis, indicated by the curved arrows. The cell number remains constant. [Color figure can be viewed at wileyonlinelibrary.com]

The process of exchanging cells with their neighbour's position in a controlled direction, whilst maintaining the cell number constant is referred to as cell intercalation.^47^ CCM can leave the tubule diameter constant, such as in tip budding during normal prostate organogenesis or lead to a reduction of the epithelial structure diameter.^48^ The latter mechanism, referred to as convergent extension (CE), is a mechanism that primitive tubules use to extend themselves, combining both collective cell migration and cell intercalation^49^, ^50^ (Figure 5). During CE, cells undergo CCM along the proximal‐distal axis where a leading edge with protrusions is formed, that is, extension, and they also intercalate and reposition themselves along the medial‐lateral axis, leading to narrowing, that is, convergence. Cell intercalation along the medial‐lateral axis is predominantly dependent on the PCP pathway.^47^

*In vitro* tumour models have shown that EMT, collective cell migration and cell intercalation can all occur in PCa migration.^51^, ^52^ Clinicopathologically, invasive cribriform architecture is an aggressive GP4 growth pattern with independent adverse prognostic value for metastasis‐ and disease‐specific‐free survival.^53^ Invasive cribriform GP4 is a contiguous epithelial proliferation with many cells not contacting adjacent stroma, generally with well‐demarcated rounded borders and lack of irregular protrusions. The absence of irregular protrusions leads us to hypothesise that migration and expansion of cribriform structures do not occur using EMT, but through collective cell migration. Collective cell migration could also represent an essential mechanism for vascular invasion, since intravascular PCa is identified as solid tumour clusters rather than single cells, as would be expected in EMT.

## Lumen expansion in normal organogenesis

Lumen expansion can occur by the fusion of multiple lumens or by increasing the existent lumen size. Lumen size is primarily regulated by apical‐basal and planar cell polarity complexes.^25^ Rho‐GTPases can also contribute to lumen size regulation through vesicle trafficking and cell shape modification.^13^ Coalescence of multiple intercellular lumens can be achieved by the slow process of vesicle trafficking, supplemented by the contraction of the actomyosin cytoskeleton.^13^ Lumen expansion is also regulated by ionic transport, and continued vesicle exocytosis increases luminal hydrostatic pressure. Pathologically, the spatial transitions of GP5 cords and poorly‐formed GP4 could be explained by active lumen formation and expansion dynamics.

## Tubular branching in normal organogenesis

**Figure 6:**
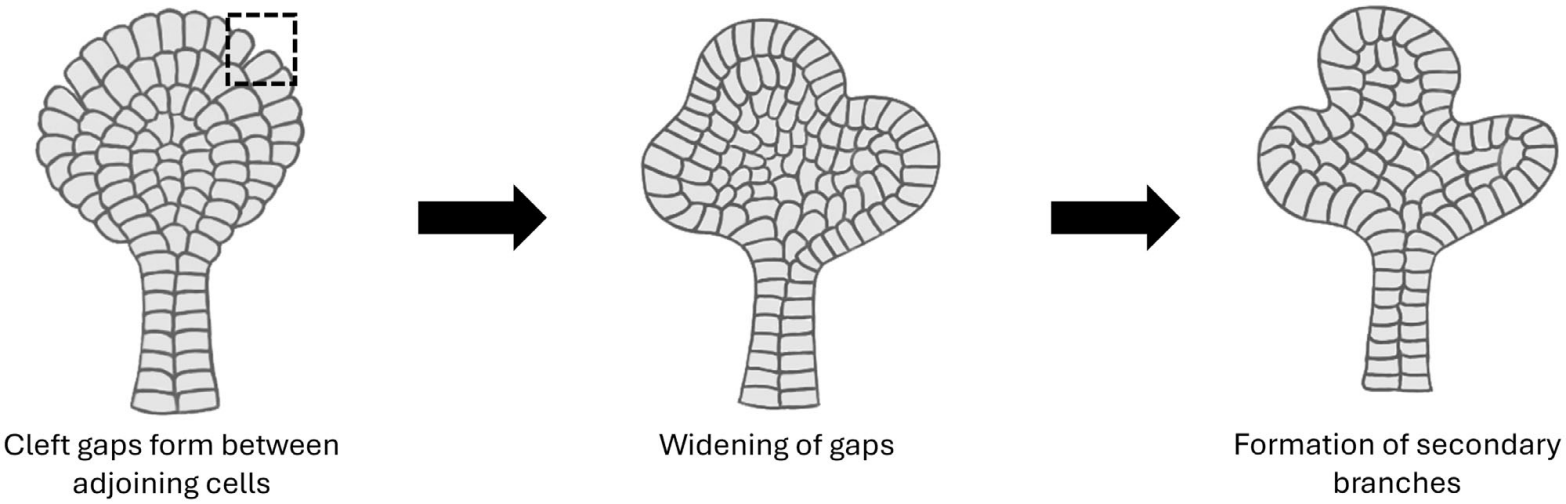
Clefting as a branching mechanism. Clefting is initiated by the formation of gaps between adjoining cells, indicated by the dotted square. The gap further widens, which eventually leads to the formation of secondary branches.

Tubular branching is essential for the development of many organs, such as lungs, mammary glands, and kidneys.^35^ Budding, cord hollowing, and cavitation are commonly used for branching morphogenesis.^25^, ^54^ Tubular branching can happen by budding and clefting of the primary tubule.^5^, ^55^ During lung and mammary gland development, a local increase in cell proliferation and collective cell migration leads to budding at the tip or lateral branching, resulting in outgrowth and elongation of secondary tubules.^56^, ^57^, ^58^ On the other hand, localized pulling of cell‐matrix contacts and cellular reorganization can lead to clefting of preexistent tubules (Figure 6). This process may be followed by proliferation, such as in the case of mouse lung and kidney, or may not involve proliferation, as seen in mouse salivary glands.^58^ Clefts originate as gaps between adjoining epithelial cells and are further stabilized by the formation of cell‐matrix contacts in epithelial cells that surround the cleft.^59^ Clefting has recently been identified as a mechanism utilized during pancreatic acini development.^57^

Normal prostatic branching follows the rules of the “branching and annihilating random walks” (BARW) model.^60^ In this model, proliferative cells are present at the tip of the branches, and the ducts branch based on a random exploration of space. When a branching tip reaches the vicinity of another ductal branch, the tip gets irreversibly inactivated by differentiation, referred to as tip‐directed termination. While the BARW model provides an explanation for developmental branching and organ definition, it might also explain an intriguing and commonly observed pathological feature in human PCa, that is, the fact that invasive adenocarcinoma does not seem to destroy and replace preexisting glands but is situated and growing in between those glands.

Using 3D reconstruction of clinical PCa, Verhoef *et al*. have shown that GP3 to five epithelial structures branch and interconnect.^4^ Whereas branching and interconnections are relatively rare in GP3, they are abundantly present in fused GP4. We therefore hypothesise that fused the GP4 growth pattern results from excessive localized budding. On the other hand, aberrant clefting might underly one of the most intriguing growth patterns in PCa, that is, glomeruloid architecture. Here, intraluminal epithelial protrusions either with or without subtle fibrovascular cores occur within a dilated GP3 tubular network frequently at places of 3D tubular branching.^4^ Aberrant clefting could result in inward movement of epithelial cells in close contact with stroma. Although a glomeruloid pattern can show overlapping morphology with a cribriform pattern, it is associated with better instead of worse clinical outcome in Gleason score 7 PCa patients.^61^

## Genomic alterations in aberrant growth patterns

**Figure 7:**
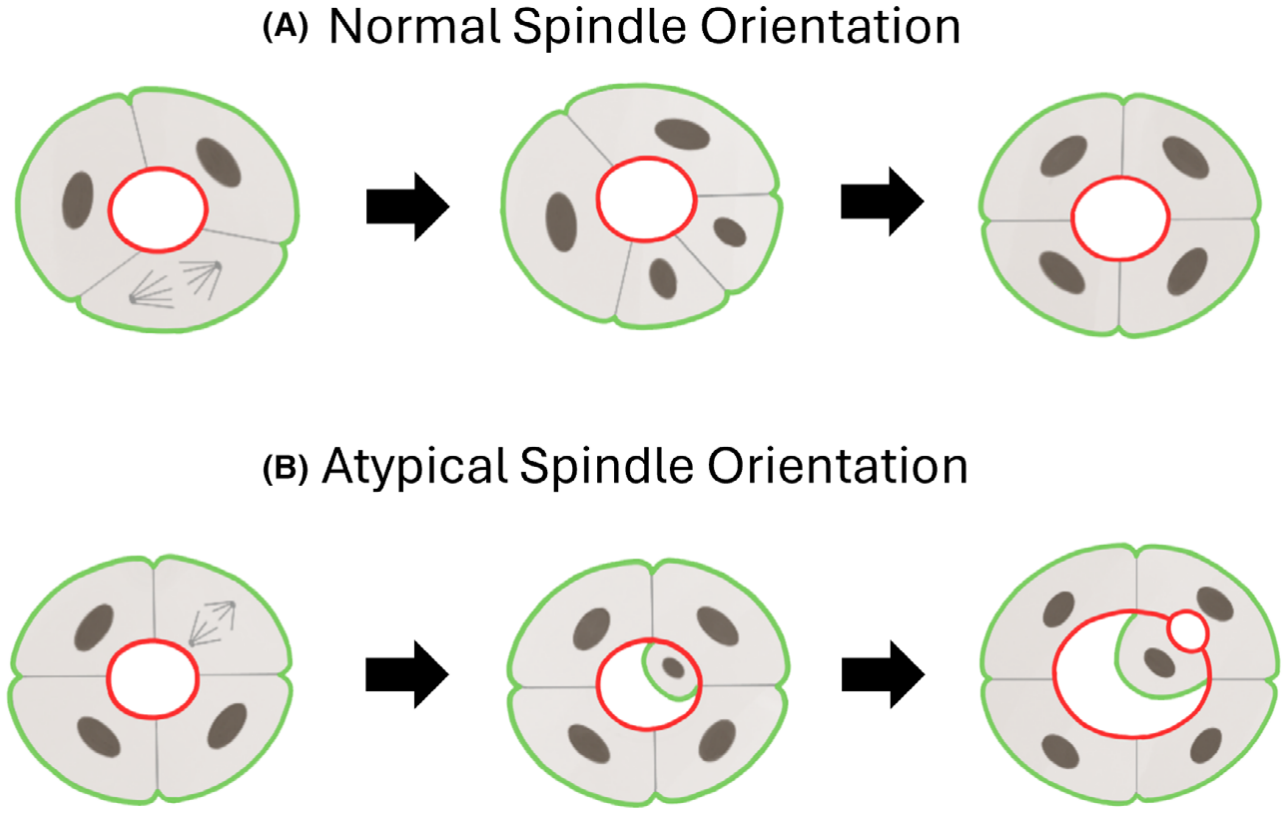
Role of mitotic spindle orientation in normal and aberrant lumen formation. The apical surface has been outlined in red and the basal in green. (**A**) Normal condition where the mitotic spindle lies perpendicular to the apical‐basal axis, that is, parallel to the central lumen. (**B**) Atypical condition where the mitotic spindle lies parallel to the apical‐basal axis, resulting in daughter cell misplacement and aberrant lumen formation. Figure adapted from Overeem *et al.*.^62^ [Color figure can be viewed at wileyonlinelibrary.com]

Within this review, we have provided hypotheses on the dynamic pathophysiological interpretation of several histopathological features in clinical PCa, such as for poorly‐formed and fused GP4, and GP5 cords and single cells. However, it is more challenging to understand the morphogenesis of some other patterns, such as cribriform GP4 and solid GP5 in the frame of established physiological developmental programs. Genomic alterations could account for aberrant growth patterns through their interference with physiological programs. For instance, Deevi *et al*. showed that *PTEN* knockdown in nonmalignant Caco‐2 colorectal epithelial cells resulted in mitotic spindle cell misorientation (Figure 7).^63^ Misalignment of those mitotic spindles gave rise to daughter cells placed on top instead of next to each other, resembling cribriform epithelial architecture. Since *Pten* knockout mice develop atypical cribriform prostatic lesions and *PTEN* loss is frequently observed in human cribriform PCa, it is tempting to speculate that such a mechanism could also explain the morphogenesis of this aggressive clinical growth pattern as well as solid GP5, which often shows spatial continuity with cribriform GP4.^64^, ^65^

## Conclusion

In the last decade, it has become clear that the assessment of individual growth patterns has independent prognostic value in PCa patients. In particular, the presence of a cribriform pattern reflects aggressive potential, while tumours without a cribriform pattern hardly ever metastasise.^38^ Understanding the clinical and molecular‐biological features of individual growth patterns is far more comprehensible than merely considering Gleason scores. Three‐dimensional imaging has revealed the actual architecture of the growth patterns, where routine pathological slides only provide a two‐dimensional cross‐sectional representation of those structures. However, knowledge of the four‐dimensional dynamics of human PCa growth patterns is still lacking.

In this review, we summarized developmental mechanisms involved in primitive tubule formation, tubule elongation, lumen expansion, and tubular branching in organogenesis. We have linked these processes to microscopical PCa pathology, hypothesising how several static features encountered in daily pathological practise could be explained from a developmental point of view (Table 2). Due to a lack of models for studying PCa growth patterns *in vitro*, gaining definitive evidence of spatiotemporal tumour dynamics will remain challenging. This study could provide a rationale for the discerned pathological interobserver variability and the clinical outcome differences between PCa growth patterns.

**Table 2:** Overview of hypothesized morphogenetic mechanisms underlying prostate cancer growth patterns and their clinicopathological significance

Growth pattern	Morphogenetic mechanism	Significance
GP3	Well‐formed tubules of polarized cells with rare branching	Minimal, if any, potential to metastasise
GP4 Poorly‐formed	Dynamic structures undergoing partial EMT and lumen formation by cord hollowing; since EMT is reversible, potential to mature into GP3 tubules?	Minimal potential to metastasise in the absence of a cribriform pattern
Fused	Well‐formed tubules of polarized cells with excessive branching and interconnections; overactivation of budding?	Simple fused resembles GP3 with branching resulting in grading variability; large complex fused resembles cribriform and has metastatic potential
Glomeruloid	Poorly understood growth pattern; caused by aberrant clefting?	GP4 with best outcome; large glomeruloid can overlap with cribriform pattern
Cribriform	Aberrant pattern with cells surviving without stromal contact caused by disturbance of mitotic spindle orientation, ex, due to *PTEN* loss?	Strongly associated with metastasis; cellular ability to survive without stromal contact relevant at metastatic sites?
GP5 Cords	Cord hollowing results in formation of minute intercellular lumens	GP5 cords with cord hollowing overlap with poorly‐formed GP4 resulting in grading variability
Single cells	Rare growth pattern with loss of cell contacts due to genomic alterations in E‐cadherin and cell hollowing	Cell hollowing should be distinguished from intercellular lumen formation observed in poorly‐formed GP4
Solid	Aberrant pattern with cells surviving without stromal contact caused by disturbance of mitotic spindle orientation, ex, due to *PTEN* loss?	Presence of minute intercellular lumens merges with cribriform pattern 4; rarely observed without cribriform pattern

EMT, epithelial‐mesenchymal transition; GP growth pattern.

## Conflict of interest

None reported.

## Data Availability

Data sharing not applicable to this article as no datasets were generated or analysed during the current study.
